# A new telomerase RNA element that is critical for telomere elongation

**DOI:** 10.1093/nar/gkt514

**Published:** 2013-06-19

**Authors:** Nancy Laterreur, Sébastien H. Eschbach, Daniel A. Lafontaine, Raymund J. Wellinger

**Affiliations:** ^1^Department of Microbiology and Infectious Diseases and ^2^Department of Biology, RNA Group, Université de Sherbrooke, 3201, rue Jean-Mignault, Sherbrooke J1E 4K8, Canada

## Abstract

The stability of chromosome ends, the telomeres, is dependent on the ribonucleoprotein telomerase. *In vitro*, telomerase requires at least one RNA molecule and a reverse transcriptase-like protein. However, for telomere homeostasis *in vivo*, additional proteins are required. Telomerase RNAs of different species vary in size and sequence and only few features common to all telomerases are known. Here we show that stem-loop IVc of the *Saccharomyces cerevisiae* telomerase RNA contains a structural element that is required for telomerase function *in vivo*. Indeed, the distal portion of stem-loop IVc stimulates telomerase activity *in vitro* in a way that is independent of Est1 binding on more proximal portions of this stem-loop. Functional analyses of the RNA *in vivo* reveal that this distal element we call telomerase-stimulating structure (TeSS) must contain a bulged area in single stranded form and also show that Est1-dependent functions such as telomerase import or recruitment are not affected by TeSS. This study thus uncovers a new structural telomerase RNA element implicated in catalytic activity. Given previous evidence for TeSS elements in ciliate and mammalian RNAs, we speculate that this substructure is a conserved feature that is required for optimal telomerase holoenzyme function.

## INTRODUCTION

Telomeres at the ends of eukaryotic chromosomes are composed of specific repeat sequences that cannot be completely replicated by the conventional DNA replication machinery (1). This shortcoming is solved, in virtually all eukaryotes, by the action of the ribonucleoprotein enzyme telomerase, a chromosome end-dedicated reverse transcriptase (RT) (2–4). The catalytic core of telomerase consists of a protean catalytic RT moiety [called TERT in mammals (5–7), Est2 in yeast (8,9)] and an RNA molecule, part of which is used as template for telomeric repeat addition [TR in mammals (10,11), Tlc1 in yeast (12)]. For the telomerase-dependent telomere lengthening reaction to occur, the enzyme must align precisely with its substrate, the telomere, on the terminal single-stranded DNA 3′-end such that part of the telomerase RNA templating region is base-paired with the DNA (13). Furthermore, the catalytic subunit must have access to this 3′-end and reverse copy the RNA sequence up to a predetermined position, which is established by a double-stranded template boundary element in the RNA (14–16). Another conserved and essential feature in the telomerase RNAs is a particular pseudo-knot structure in the catalytic center (17–22).

**Figure 1.**
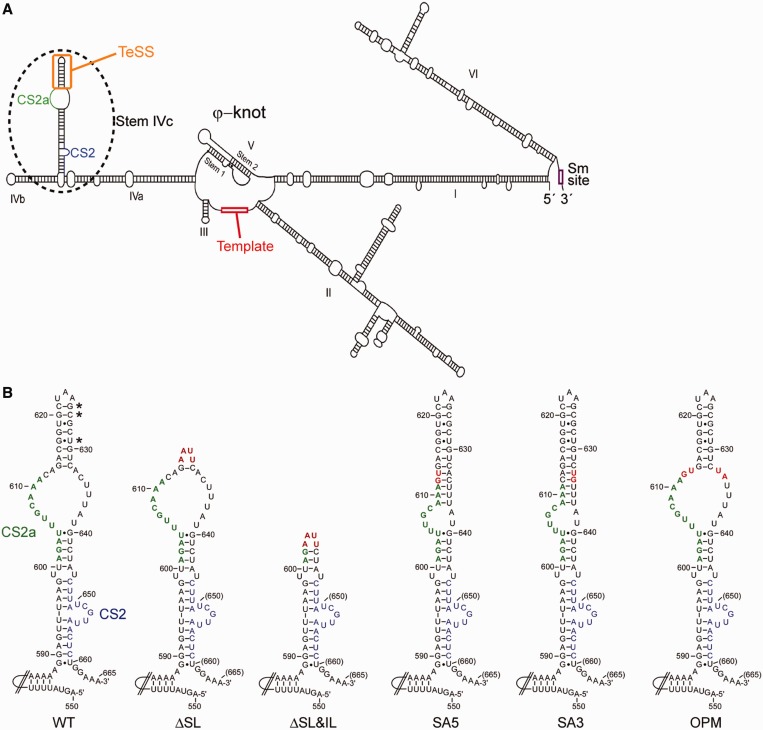
Predicted structures of Tlc1 RNA variants. (**A**) Schematic for the predicted overall secondary structure of the budding yeast telomerase RNA Tlc1. Important features such as the Sm-binding site near the 3′-end, the template (in red) the pseudo-knot and stem IVc (dashed oval) are highlighted. Colored elements on stem IVc are the CS2 element (in blue), the CS2a element (in green) and the TeSS (in orange). (**B**) MFold predicted secondary structures of the stem IVc arm of the Tlc1 RNA and key subelements. Blue: CS2 element. Green: CS2a element as in (A). Stars indicate co-varying base pairs. Red: positions of the mutated nucleotides in the different constructs.

In addition to those conserved elements, species-specific sequences ensure its stability and correct trafficking (2). The budding yeast *Saccharomyces cerevisiae* telomerase RNA is predicted to fold such that three relatively long arms emanate from the catalytic center [(17,18); Figure 1 and Supplementary Figure S1]. These substructures have been compared with functional domains as at least some of them can be permuted on the RNA without complete loss of activity (17,23). One of these arms ends in a short stem-loop that is associated with binding to the yeast Ku proteins (24). This Ku–Tlc1 RNA interaction is important for telomerase import and/or retention in the nucleus but does not appear to impinge directly on catalytic activity (25). In contrast, a substructure of another arm, forming a bulged stem-loop between nucleotides (nt) 589 and 660 of the Tlc1 RNA around the conserved sequence 2 (CS2) element associates with Est1, an essential protein subunit for telomerase activity *in vivo*, but not *in vitro* (26–29) (Figure 1 and Supplementary Figure S1). The Est1 protein tethers telomerase to telomeres as it interacts with Cdc13, a protein that is bound on the terminal single-stranded DNA occurring on telomeres (30–32, reviewed in 33). This tethering, however, is restricted to late S-phase of the cell cycle, the time when telomere elongation occurs (34–36). Moreover, the idea of an Est1-mediated tethering of telomerase to telomeres is supported by experiments in which the Cdc13 protein is fused to the catalytic subunit of telomerase Est2. In this setting, Est1 in fact becomes dispensable and the fusion protein will allow functional telomerase-dependent telomere maintenance (32).

We recently began investigating the Est1–Tlc1 RNA interface in more detail. The approach was prompted by our previous finding of a new and conserved sequence element called CS2a (37) on the telomerase RNAs of *Saccharomyces*, *Kluyveromyces* and *Candida spp.* near the previously reported budding yeast Est1 binding site (CS2; Figure 1). Preliminary analyses showed that CS2a appeared essential for telomere maintenance *in vivo* (37,38). Here, our results show that CS2a is essential for Est1 association with the telomerase RNA and the intranuclear functions of telomerase, but dispensable for import of telomerase from the cytoplasm. More surprisingly, a targeted analysis of the predicted structure distal to the CS2/CS2a region, an internal bulge followed by an apical hairpin, indicates that this latter domain of the RNA is set apart of the Est1 site. The sequences on this part of the RNA are not conserved, but the apical stem has several co-varying base pairs, indicating structural conservation. Furthermore and most importantly, it needs to be flanked by the flexible internal bulge for efficient telomerase function. Loss of the distal stem-loop or mutations that cause a partial closing of the flexible internal loop (IL) cause telomere shortening. In addition, telomerase activity isolated from strains harboring these mutations in *TLC1* is reduced by ∼50%, irrespective of whether Est1 was present or not. Taken together, these results indicate that the apical architecture of stem IVc ranging from nt 612 to 637 (Figure 1), a substructure we call telomerase-stimulating structure (TeSS) in the budding yeast telomerase RNA is critical for *in vivo* catalytic activity in a way that is independent of the Est1 protein. We propose that an induced fitting to the catalytic center of a distant short stem-loop on telomerase RNAs is a new conserved feature in the mature telomerase complexes of many species.

## MATERIALS AND METHODS

### Strains and plasmids

Strain NLYH80 containing plasmid pAZ1 was used as base strain for most experiments (Supplementary Tables S1 and S2). The plasmid pTLC1TRP (39) was used as a base vector for introducing the described mutations. For telomerase assays, strains NLYH80 (*EST1*) and NLYH95 (*est1Δ*) with the indicated Tlc1 RNA constructs were used. The *CDC13* gene disruptions in strains NLYH55 and NLYH59 were made by replacing the genomic locus by the *KanMX* cassette using a one-step polymerase chain reaction (PCR)-mediated method (40). The deletion of *YKU70* in strain NLYH97 was carried out as above. *EST1* deletions were achieved using a LoxP-*KMX*-LoxP cassette (41). To recycle the KMX marker, the Gal-Cre fragment from pSH62 (41) was subcloned into pRS316 (EuroScarf) and, following induction of the Cre recombinase by galactose, clones were verified for excision of the *KMX* marker on growth and replica-plating on Sc-Ura and Sc-Ura + G418 plates, respectively. Loss of the pRS316-Gal-Cre plasmid was then carried out on 5-Fluoroorotic Acid (5-FOA) plates and the final strain containing the *est1Δ::LoxP* allele was verified by PCR and Southern blotting. *TLC1* deletion in NLYH55, NLYH59 and NLYH95 was done by replacing the genomic locus by the NatR resistance cassette. pVL1107 (32) was a kind gift of Victoria Lundblad. The HA_3_-*EST1* construct was obtained as in (42) and tagging of *SME1* with the Myc_13_ epitope was achieved by a PCR-based gene targeting using the pFA6a-13Myc-KMX plasmid (43). Clones were verified by PCR, Southern and western blotting. *TLC1* variants SA5, SA3, OPM, OPC and apical loop disruption mutants were made by a PCR-mediated site-directed mutagenesis using the primers described in Supplementary Table S3 using the pTLC1TRP vector as a base. Note that the OPC allele was created by performing the PCR-mediated mutagenesis on the SA5 construct using the SA3 primers. Plasmid pAD-A258 (*tlc1Δ148-440*) was constructed by replacing the wild-type (WT) *TLC1* allele of the *URA3*-based vector pADCEN36 (18) by the *tlc1Δ148-440* allele (27). For *EST1* overexpression studies, plasmids pRS423 (control) or pRS423-*EST1* were transformed into yeast strain NLYH80 expressing the various *TLC1* variants.

### Synthetic interaction with *yku70Δ*

Cells of strain NLYH80 (*YKU70*) or NLYH97 (*yku70Δ*) expressing the various *TLC1* alleles were grown to an OD_660_ of 0.7 and spotted in serial dilutions on selective SC-Trp-Ura and FOA-Trp media to test for viability after loss of the complementing WT *TLC1* plasmid (pAZ1).

### Telomere length analyses

After selection for cells that had lost pAZ1(*TLC1* WT) on 5-FOA plates, cells from indicated passages were grown in liquid media. Genomic DNA was extracted, digested with XhoI, subjected to agarose gel electrophoresis (0.75% agarose), transferred to a nylon membrane and hybridized to a 300 bp fragment containing 280 bp of telomeric repeats derived from pYLPV (44). Probes for hybridization were obtained by random priming labeling procedure (45). Data were visualized and analyzed using a Typhoon FLA9000.

### Northern blot analyses

Total RNA was extracted from 10-ml cultures of exponentially growing cells. After resuspension of RNA pellets in nuclease-free water, northern blot analysis was performed as previously described (46). Briefly, 5–10 μg of total RNA was run on a 1% MOPS-agarose gel and transferred on a Hybond-N+ membrane (GE Healthcare, Canada). RNA was visualized using a probe specific for *TLC1* (a 686-bp NcoI-NsiI restriction fragment). The membranes were rehybridized with *U1*- and *ACT1*-specific probes for loading control and quantification. Analyses of RNA levels were performed using a Typhoon FLA9000 apparatus and the Quantity One software.

### Protein extraction, immunoprecipitations and telomerase assay

Inside cells, telomerase RNA is bound a few nucleotides upstream of its mature 3′-end by the Sm_7_ complex, and this interaction is essential for Tlc1 RNA stability (47). We therefore argued that SM-proteins could be useful targets for immuno-enrichment of telomerase, without disturbing its catalytic center. For the telomerase assays, we thus introduced all the above alleles of *TLC1* as the sole *TLC1* gene into a strain that also harbored a Myc_13_-tagged Sme1 protein. Total protein extracts were prepared using a slightly modified protocol of that described in (48). Briefly, 500 ml of cells grown to an OD_660_ of 1.0 were pelleted, washed once with cold water and once with TMG buffer (10 mM Tris–Cl, pH 8.0, 1 mM MgCl_2_, 10% glycerol) supplied with 200 mM NaCl. The cell pellets were then frozen in liquid nitrogen and lysis was performed by grinding the pellets in presence of pieces of dry ice in a standard coffee mill (Krups). The cell powder was thawed on ice and one pellet volume of TMG buffer with 200 mM NaCl, 0.1 mM DTT, 0.2% Triton X-100, 0.2% NP40 and protease inhibitors was added. An S-100 was performed in an ultracentrifuge for 2 h at 100 000*g* (4°C). Immunoprecipitations (IP) used 2 mg of total proteins adjusted to 0.5% Tween-20 and to which 40 U of RNasin per ml of extract (Promega, USA) was added. Following an incubation of 2–3 h at 4°C with 500 ng of anti-MYC antibody (mouse monoclonal, clone 9E10, Roche Diagnostics, USA), 50 μl (bed volume) of TMG-washed Protein G plus/Protein A agarose (Calbiochem, USA) were added and everything was incubated overnight at 4°C with gentle agitation. Beads were then washed twice with 0.5 ml TMG plus 200 mM NaCl, 0.1 mM DTT, protease inhibitor and 0.5% Tween-20, and twice with 0.5 ml TMG plus 0.1 mM DTT, protease inhibitor and RNasin added at 40 U per 0.5 ml of TMG. Beads were resuspended in one bed volume of TMG plus 0.5 mM DTT, protease inhibitor and 40 U of RNasin. The actual telomerase activity assay was performed as previously described (48) on 5 μl bed volume of immunoprecipitation beads. Extension of a telomeric primer (Supplementary Table S3) was monitored to determine telomerase activity *in vitro*. Visualization and quantification of extension products were performed using a Typhoon FLA9000 apparatus and the Quantity One software.

### Fluorescent in situ hybridization

Cells from NLYH80 and NLYH95 were transformed with the *TLC1* variants except for the *tlc1Δ* sample, which consists of NLYH80 that has lost the complementing *TLC1* plasmid pAZ1 on 5-FOA. Yeast cultures were grown to an OD_660_ of 0.4, and fluorescent *in situ* hybridization (FISH) experiments were performed as previously described in (25,49). After fixation, cells were hybridized with Cy3-labeled probes (Supplementary Table S3), and single-molecule detection was achieved by using a wide-field epifluorescence (49) with a Zeiss® spinning disk AxioCam microscope. Results were analyzed and quantified using the FIJI platform for Image J (50).

### Selective 2′hydroxyl acylation analyzed by primer extension

WT and mutant stem-loop IVc RNA molecules were prepared as previously described (51) by adding a primer binding site downstream of the studied RNA molecule. Selective 2′hydroxyl acylation analyzed by primer extension (SHAPE) reactions were performed by using 1 pmol of RNA resuspended in one volume of 1× TE (10 mM Tris–HCl, pH 8.0, 1 mM EDTA) buffer, which was mixed with one volume of 3.3× folding buffer containing 333 mM K-HEPES, pH 8.0, 333 mM NaCl and 10 mM MgC_l2_. RNA samples were heated to 70°C, slowly cooled to room temperature and incubated 10 min at 37°C. Next, samples were reacted in presence of *N*-methylisatoic anhydride for 80 min at 37°C. Reactions were ethanol precipitated, washed with 70% ethanol, dried and then resuspended in 0.5× TE buffer. Reverse transcription reactions were performed according to the supplier’s protocol. Gels were exposed to PhosphorImager screens and imaged.

### *Est1* binding assay and RT-PCR

NLYH80 (HA_3_-*EST1*) cells were co-transformed with a plasmid harboring *TLC1* alleles (either WT or with mutations in the stem-loop IVc domain) as well as the pAD-A258 (*tlc1Δ148-440*) plasmid. The RNA expressed from the latter invariably contains the complete and WT Est1 and Est2 binding domains and thus serves as internal positive control (I.C.). Yeast cells (500 ml) were grown up to OD_660_ of 1.0. Total protein extracts and HA_3_-tagged Est1 immunoprecipitations were prepared as described in the methods for telomerase assays except that the extracts were incubated with 500 ng of a high-affinity anti-HA (3F10, rat monoclonal, Roche Diagnostics, USA) for 2–3 h at 4°C before adding the TMG-washed beads. Total RNA and post-IP flowthrough RNA were prepared as described (27). Reverse transcription was performed on 5 µl bead volume (IP), 2 µl (5%) input RNA and 2 µl (5%) flowthrough RNA. The RT Superscript II protocol (Invitrogen, USA) was followed using the RT P_0_ primer listed in Supplementary Table S3. After RT, cDNAs were purified on MinElute PCR purification columns (Qiagen, USA) and elution was performed in 17 µl elution buffer. The subsequent PCR reaction was done on 5 µl of the purified cDNA with 20 pmol of each P1-FOR and P2-REV primers (Supplementary Table S3). Detailed PCR conditions will be supplied on request. PCR products were then analyzed by electrophoresis on 1.5% agarose-TAE gel.

## RESULTS

### Structural mutations in the Tlc1 RNA that compromise telomere maintenance

**Figure 2.**
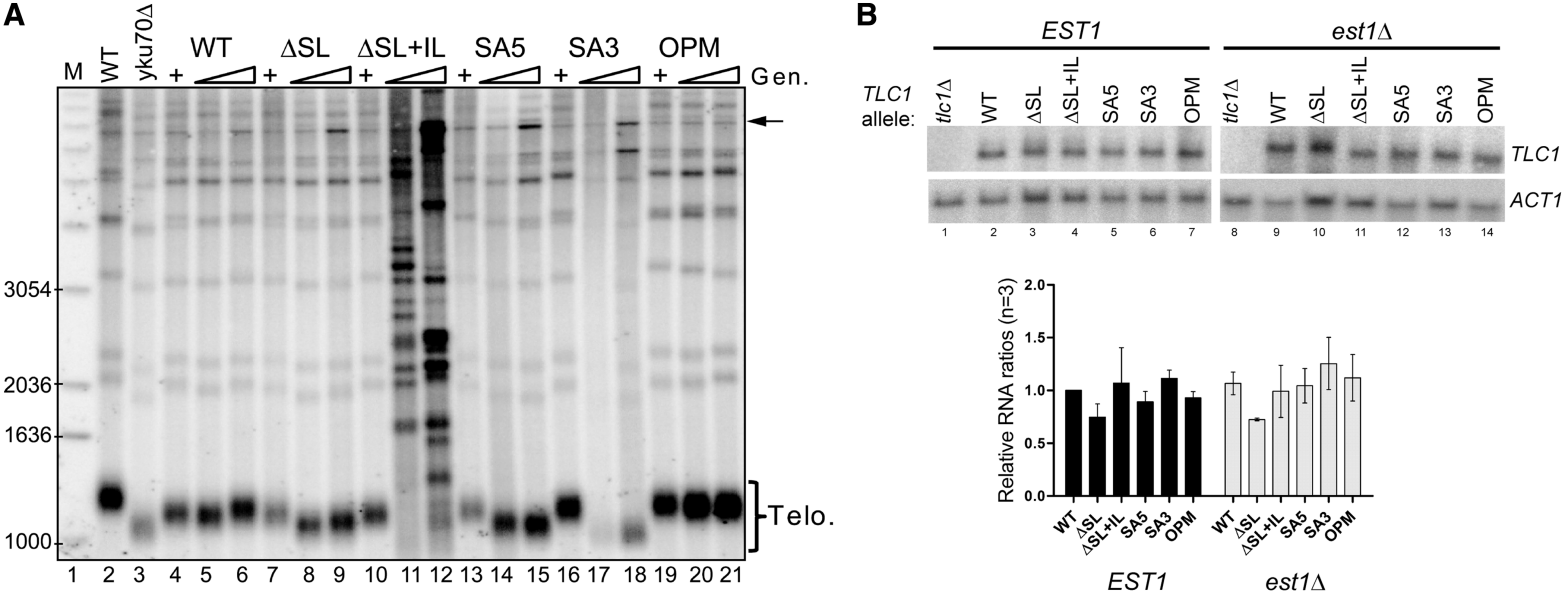
Expression levels and telomeric phenotypes in cells expressing Tlc1 variants. (**A**) Telomere length analysis of cells harboring the mutated Tlc1 RNAs. Lane 1: (M) Radiolabeled 1 kb DNA ladder with selected sizes indicated on left of gel. Lanes 2 and 3: WT and *yku70Δ* controls. Lanes 4, 7, 10, 13, 16 and 19 (+): cells expressing the indicated *TLC1* allele plus the WT *TLC1* complementing plasmid (pAZ1). Lanes 5, 8, 11, 14, 17, 20: cells with only the indicated *TLC1* allele were grown for 105 generations. Lanes 6, 9, 12, 15, 18, 21: same strains grown to 205 generations. Terminal restriction fragments (Telo) and amplified Y′ elements (arrow) are indicated on the right. (**B**) Northern blot analysis of RNA derived from NLYH80 (*EST1*, left) and NLYH95 (*est1Δ, *right) cells expressing WT or indicated mutant Tlc1 RNAs. Lanes 1: RNA extracted from NLYH80 cells that had lost the complementing *TLC1* plasmid pAZ1 (*EST1 tlc1Δ *cells); lane 8: RNA extracted from NLYH95 cells that had lost the complementing *TLC1* plasmid pAZ1 (*est1Δ tlc1Δ *cells); lanes 2–7: RNA from NLYH80 cells expressing indicated *TLC1* alleles; lanes 9–14: RNA from NLYH95 cells expressing indicated *TLC1* alleles. Top panels: membrane hybridized with the *TLC1* probe. Bottom panels: same membrane rehybridized with the *ACT1* probe for loading controls and quantification. Below the gels, graph of the quantification of relative RNA levels from three independent experiments. Tlc1 RNA level was normalized against that of Act1 RNA and compared with the RNA levels in the WT strain.

Stem-loop IVc of the budding yeast telomerase RNA is involved in Est1 binding. In fact, sequences around CS2 [nt 646–659, blue on Figure 1; (26)] and CS2a [nt 601–611, green on Figure 1; (37)] are essential for Est1p association with the RNA (38) as well as for telomere maintenance *in vivo* (37) (Figure 2A). However, a mutation affecting only the distal-most stem-loop, the *tlc1-ΔSL* allele (Figure 1B and Supplementary Figure S1), and that is not predicted to interfere with these Est1-binding sites, also caused short telomeres (Figure 2A). To investigate the structural requirements of this distal stem-loop IVc in more detail, we introduced mutations that affect its overall structure without changing the length of the sequence. In the alleles SA5 and SA3, two nucleotides were changed such that the IL becomes more closed, essentially creating an extended distal stem-loop (Figure 1B, changed nucleotides in red). In two other alleles, the same positions in the IL were changed in a way not to allow canonical base pairing, as in the WT, thus reestablishing a same size IL but with altered sequence (OPM and OPC, Figure 1B and Supplementary Figure S1). To ensure that the mutations did not completely disrupt the overall structure of stem IVc, we probed the local nucleotide flexibility by SHAPE (52,53) using *in vitro* transcribed RNAs (Supplementary Figure S2). This technique is particularly relevant to discriminate flexible and reactive regions from relatively constraint RNA regions, such as base-paired stems for example. On all RNAs, the predicted apical loop (nt 623/624) displayed strong reactivity, consistent with their unconstrained position in the loop, which causes clearly visible bands on the gels (Supplementary Figure S2, A623/A624). This result confirms that the loop nucleotides in the terminal hairpin remained unaffected by any of the mutations. On the WT and the OPM RNAs, nucleotides of the IL (C608 to G614 on Supplementary Figure S2) also reacted well, yielding the predicted reaction pattern. In contrast, in the SA5 and SA3 mutant RNAs, these same nt 608–614 did not react, resulting in low band signals. This result is consistent with the prediction that in the mutant RNAs, the internal bulged area is more constrained by base pairing, i.e. the distal hairpin, which in the WT and OPM RNAs comprises nt 614–632, is extended in the SA3 and SA5 RNAs to nt 609–637 (Figure 1 and Supplementary Figure S2). We therefore conclude that at least *in vitro*, these RNAs fold in ways that are consistent with the *in silico* predictions. Strikingly, despite the overall similarity of the structures, the reduction of the internal bulge size had a significant impact on telomerase function *in vivo*. In cells harboring the SA5 and SA3 alleles, telomeres are extremely short (Figure 2A, lanes 14, 15 and 17, 18), and these cells failed to grow at 37°C (data not shown). Furthermore, after extended outgrowth, DNAs derived from cells harboring either of the SA3 and SA5 alleles exhibited a characteristic amplification of a 6.7 kb Y′ DNA fragment (Figure 2A) that is associated with telomerase independent maintenance of telomeric repeat sequences (33). In contrast, both the OPM and OPC alleles of *TLC1* that change the sequence but not the structure of the internal bulge supported WT telomere length (Figure 2A and Supplementary Figure S3). Note that the OPC allele corresponds to the combination of the SA5 and SA3 mutations, while the OPM allele had a one other nucleotide change (Figure 1B), yet both always displayed the same phenotypes that are indistinguishable from WT. For the rest of the study, we primarily used OPM as the allele for an open IL with altered sequence. Northern blot analyses showed that steady state levels of telomerase RNAs containing these mutations are much comparable (Figure 2B) and all RNAs were also expressed at levels that are similar to those detectable in cells with an unperturbed endogenous chromosomal *TLC1* locus (Supplementary Figure S4A, top). These results indicate that the shortened telomere lengths are not a result of lowered expression levels of the RNA, but rather a consequence of reduced telomerase activity.

**Figure 3.**
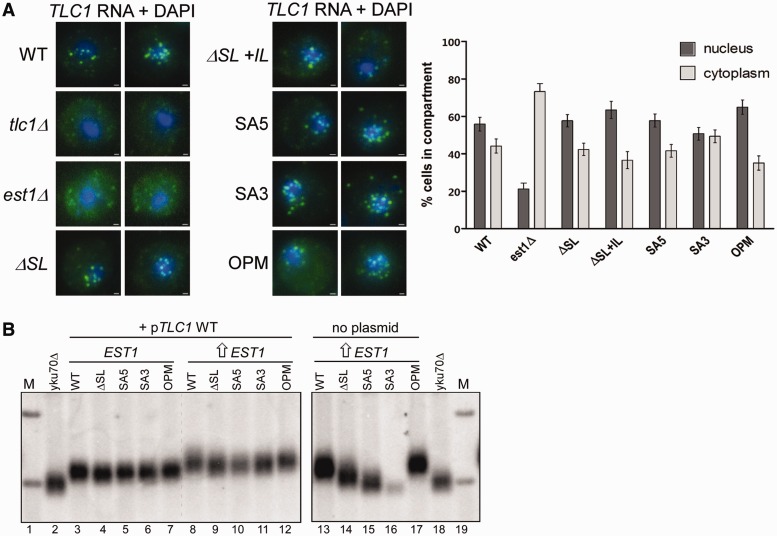
Characterization of Est1 function in conjunction with *TLC1* alleles. (**A**) Top: FISH assay detecting the Tlc1 RNA in WT, *tlc1Δ*, *est1Δ* strains or strains that harbor the various *TLC1* alleles. The Tlc1 RNA in all cells with different *TLC1* alleles display a similar nuclear distribution. Each image is a representation of one cell from the corresponding strain and the signals from both DAPI (blue, DNA) and Cy3 (green, Tlc1) channels were merged. Scale bar = 500 nm. Bottom: graphical representation of cellular localization from 50 cells of each WT, *est1Δ*, *Δ*SL, *Δ*SL + IL SA5, SA3 and OPM strains. (**B**) Overexpression of Est1 does not recapitulate telomere elongation in the *Δ*SL, SA5 and SA3 mutants. Genomic DNA from NLYH80 (+ indicated *TLC1* alleles) was extracted after 50 generations of growth in the following conditions: lanes 3–7: cells contain an empty pRS423, the complementing *TLC1* plasmid (pAZ1) and the indicated variant alleles of *TLC1*. Lanes 8–12: cells contain pRS423-*EST1* (2 µm plasmid for overexpression), the complementing *TLC1* plasmid (pAZ1) and *TLC1* variants. Lanes 13–17: cells contain pRS423-*EST1* and only the *TLC1* alleles as indicated. Lanes 2 and 18: *yku70Δ* (short telomere length control). Lanes 1 and 19 (M): radiolabeled 1 kb DNA ladder.

**Figure 4.**
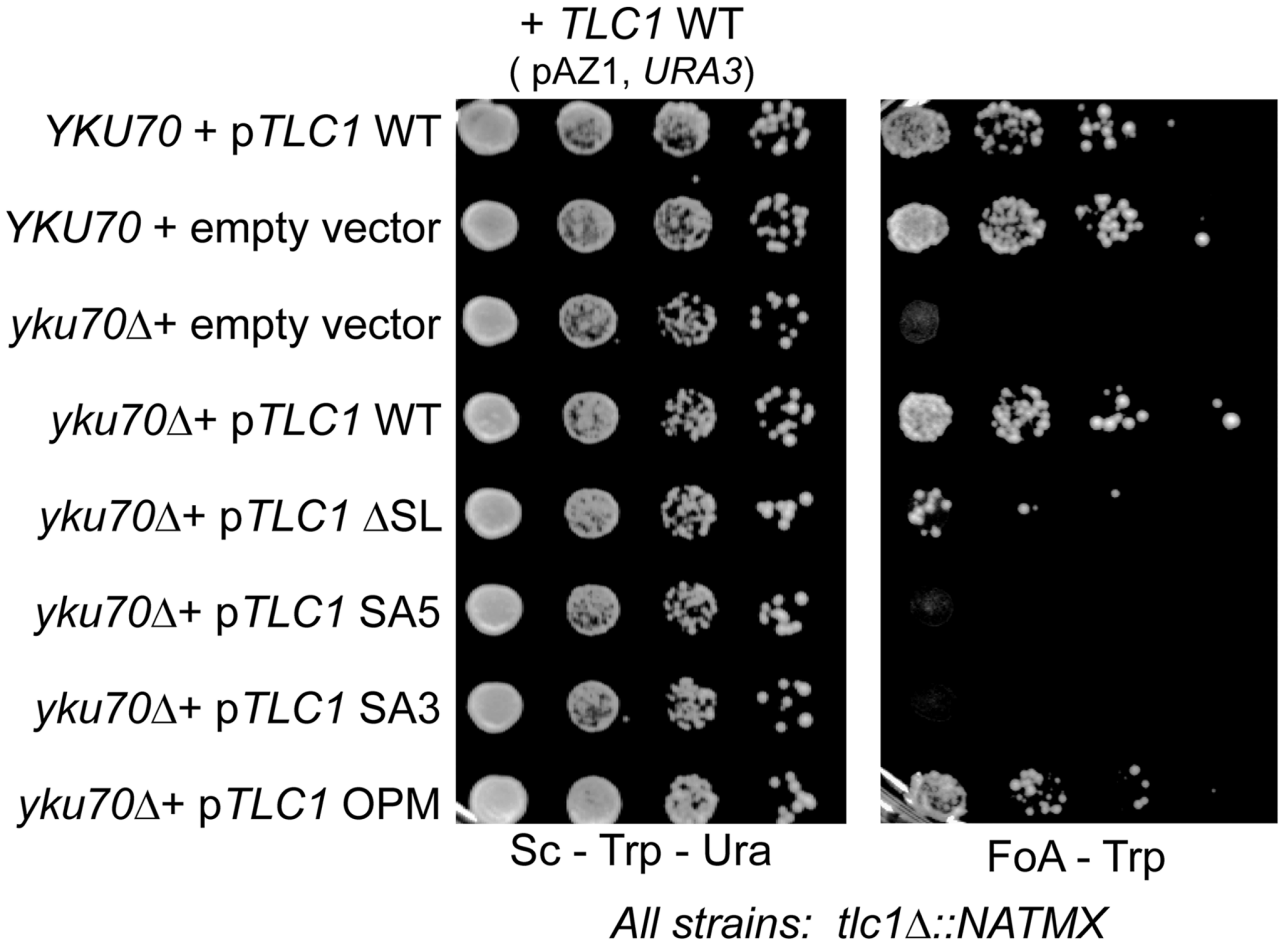
SA5 and SA3 Tlc1 RNA variants display a synthetic interaction with *YKU70*. NLYH80 (*YKU70*) and NLYH97 (*yku70Δ*) strains were grown to exponential phase and after 10-fold serial dilutions, cells were spotted on the indicated selective media. Left plate: Sc-Trp-Ura media allows cells to grow with both the different versions of p*TLC1* (*TRP1*) or the empty vector (pRS314-*TRP1*) together with the complementing *TLC1* plasmid (*URA*). Right plate: FOA-Trp media for eviction of the WT copy of *TLC1* (pAZ1).

Given the proximity of the SA3 and SA5 mutations to the Est1-binding elements CS2a and CS2, we examined whether the mutated RNAs displayed deficiencies in Est1-mediated functions such as telomerase import from the cytoplasm into the nucleus. As assessed by FISH, the majority of the signals for WT Tlc1 RNA as well as for all tested mutant alleles localized to the nucleus (25), while in the absence of Est1, there is a reversal of localization and the Tlc1 RNA now is mostly in the cytoplasm (Figure 3A, Supplementary Figure S4B). We conclude that telomerase trafficking into the nucleus is not affected by the ΔSL, SA5, SA3 or the OPM mutations (Figure 3A). Consistently, a semiquantitative co-immunoprecipitation assay also confirmed that Est1 could associate with the *tcl1-ΔSL* allele, confirming an earlier report [(38); Supplementary Figure S4C], while the SA5 and SA3 alleles displayed a reduced but detectable association with Est1 (data not shown). More importantly, while overexpression of Est1 could lengthen telomeres in WT cells (26) or cells expressing the OPM Tlc1 RNA (Figure 3B left, lanes 8–13 and 17), it failed to rescue the short telomere phenotype in cells expressing SA5, SA3 or ΔSL mutant versions of the Tlc1 RNA (Figure 3B, right, compare lanes 14–16 with 13 or 17). Cells devoid of the yeast Ku proteins are sensitive to losses of telomerase activity and display synthetic lethal interactions with deletions of telomerase subunits (54). We therefore assessed potential genetic interactions of the new *TLC1* alleles with loss of Ku (*yku70Δ*). Consistent with an important decrease of telomerase activity conferred by the mutations *in vivo*, none of the SA3, SA5 or ΔSL mutations could support growth in the absence of Ku, while the OPM allele did not show a synthetic interaction with *yku70Δ* cells and cells with that version of the Tlc1 RNA grew as WT (Figure 4).

### An Est1 independent loss of telomerase activity

**Figure 5.**
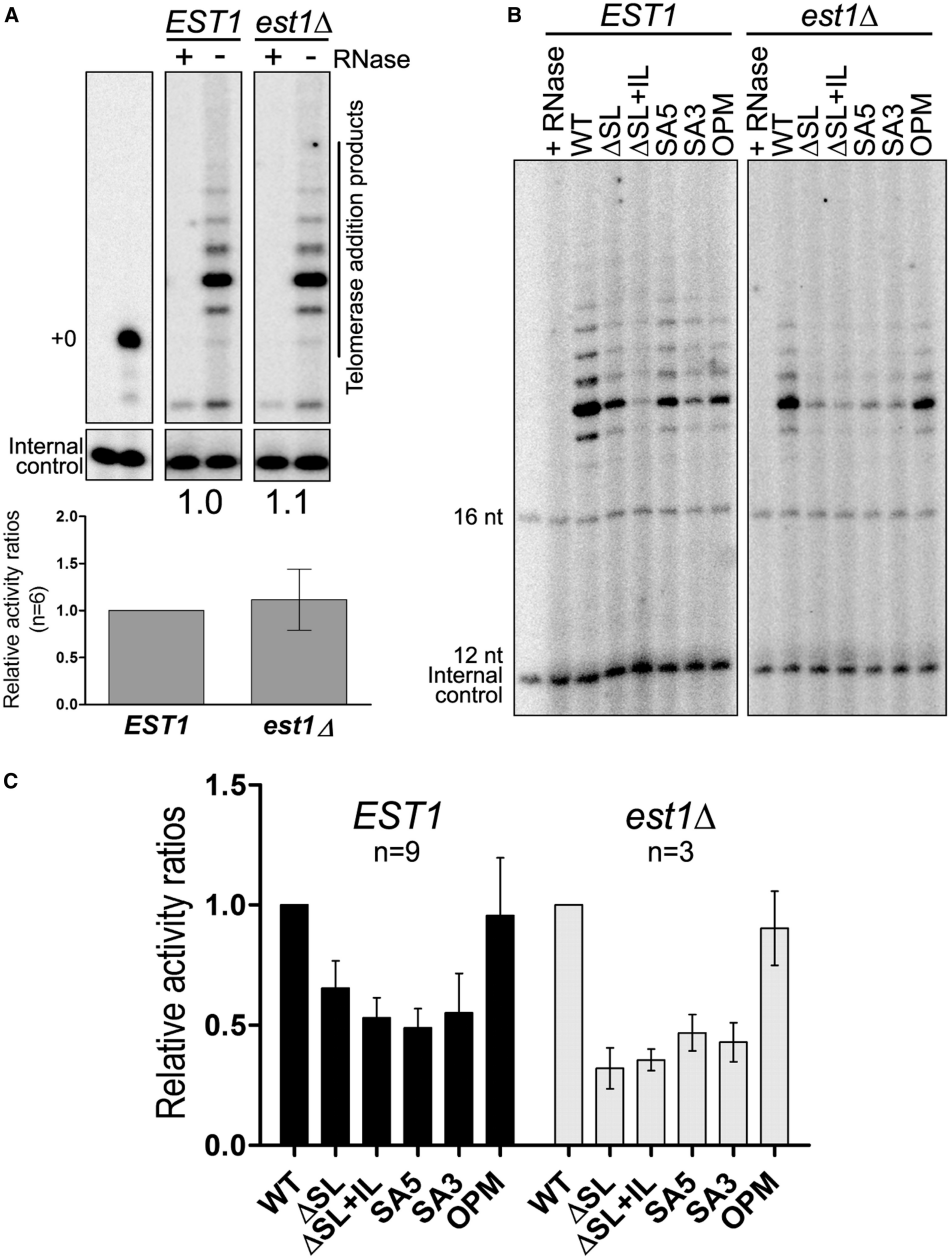
Tlc1 RNA variants show an Est1-independent decrease in telomerase activity. (**A**) Sme1-Myc_13_ immunoprecipitates from strains NLYH80 (*EST1*, p*TLC1* WT) and NLYH95 (*est1Δ*, *pTLC1* WT) were assayed for telomerase activity *in vitro* in presence of dGTP^32^ and a 16-nt telomeric primer (NLTAG1-3). Left panel: 5′-end labeled random 16-nt oligo (2000 cpm) indicates the start position. Middle panel: telomerase addition products from the *EST1* strain. Right panel: telomerase addition products from the *est1Δ* strain. Each sample had also been treated with RNase in parallel to verify RNA-dependent activity (lanes marked with +). Internal control: 5′-end labeled random 12-nt oligo, 2000 cpm. Bottom graph: relative telomerase activities derived from six independent experiments. Telomerase addition products counts were normalized with that of the internal control and compared with the telomerase activity in the WT strain. (**B**) Telomerase activity assays of the same strains as in (A), except that they were transformed with the indicated *TLC1* variants. First lane of each panel shows that the observed telomerase extension products are RNA dependent as treatment with RNase abolishes specific activity. (**C**) Graphical representation of relative telomerase activity ratios from 9 (*EST1*, dark grey) and 3 (*est1Δ,* light grey) independent experiments. The relative band intensities of the extension products were quantified and obtained values were adjusted relative to the WT Tlc1 RNA level, which was set as 1.

Because telomerase trafficking is not affected by the SA5, SA3 or ΔSL mutations but the genetic evidence suggested an important loss of telomerase activity, we assessed whether these RNAs could support catalytic telomerase activity as assayed in cell extracts from these strains. Telomerase assays were conducted using an enriched telomerase RNA fraction that was obtained after immunoprecipitation of cell extracts with antibodies directed against a Myc-tag on the Sm-protein Sme1 that is normally associated with Tlc1 RNA near its 3′-end (47). Telomerase activity itself is measured as an RNase sensitive primer extension activity (Figure 5A) in which the relative band intensities of the extension products were quantified for WT and the various mutant RNAs. Actual activity obtained with the mutant RNAs is presented relative to the WT RNA. Given that all RNA variants occur at about the same steady state level (Figure 2B), these derived values thus represent the relative efficiency of nucleotide incorporation per molecule of full-length RNA. The *est1Δ* cells did not exhibit defects in telomerase activity (Figure 5A, bottom), confirming earlier results suggesting that the Est1 protein is not required for telomerase activity as assayed from extracts *in vitro* (28,55). In stark contrast, relative telomerase activity generated by the SA5, SA3 or the ΔSL mutant version of the telomerase RNA were significantly reduced to ∼50–60% of that obtained with the WT RNA or the OPM RNA (Figure 5B and C, left parts). Virtually identical results were obtained with extracts derived from cells that were devoid of Est1 (*est1Δ-*cells; Figure 5B and C, right parts), once again confirming that effects of the introduced mutations in the Tlc1 RNA are Est1-independent. Note that as in *EST1 WT* cells, all Tlc1 RNA alleles are expressed at comparable levels in *est1Δ* cells (Figure 2B). These results thus indicate that the SA3, SA5 and ΔSL mutations directly reduce telomerase catalytic activity *per se*, without affecting the respective RNA expression levels or the Est1-dependent trafficking activities.

### The apical bulged stem-loop structure of IVc is involved in a postrecruitment step of telomerase activity

**Figure 6.**
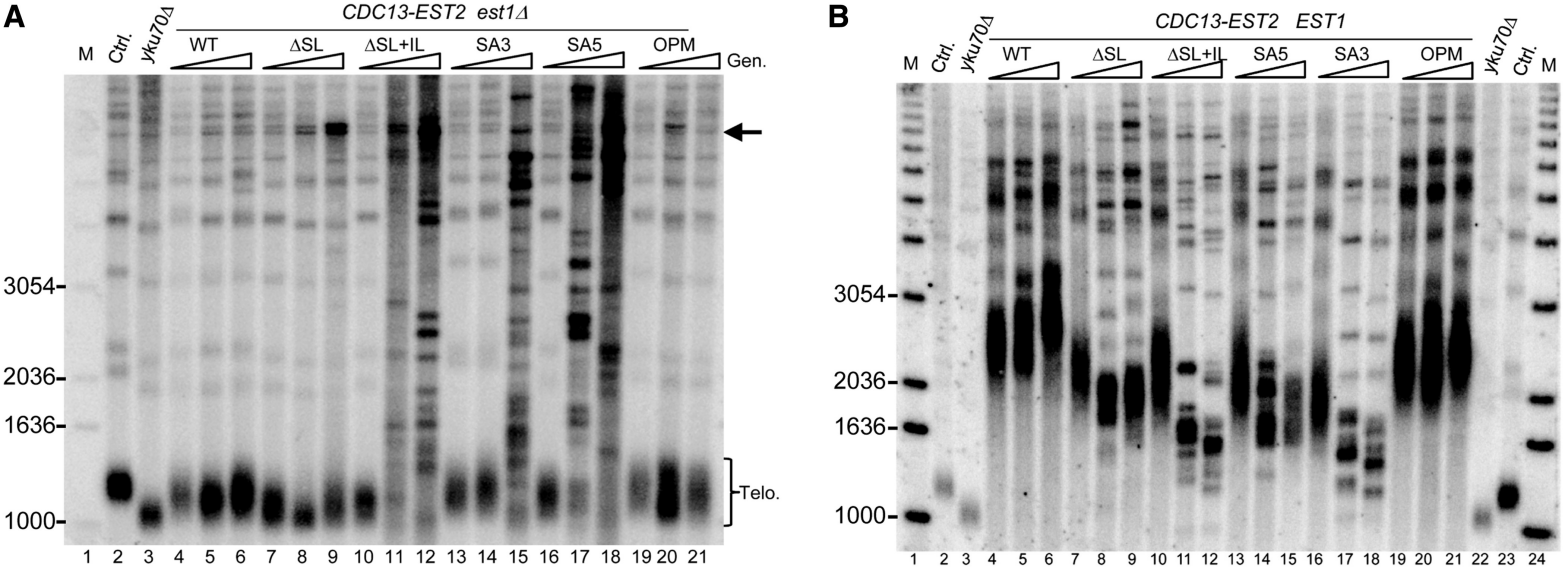
Telomere lengths in cells expressing the Cdc13–Est2 fusion. Telomere length maintenance is defective in cells with the *tlc1-SA5* and *tlc1-SA3* alleles, even when the requirement for Est1 is bypassed by the expression of a Cdc13–Est2 fusion protein. (**A**) Telomere lengths in NLYH59 (*est1Δ*) cells expressing the indicated *TLC1* alleles and the Cdc13–Est2 fusion protein. Lane 1 (M): radiolabeled 1 kb DNA ladder with selected specific sizes indicated on the left of the gel. Lane 2: WT control (SGY40). Lane 3: *yku70Δ* control for short telomeres (SGY42). Lanes 4, 7, 10, 13, 16 and 19 (+): cells expressing the indicated *TLC1* allele plus the WT *TLC1* complementing plasmid (pAZ1). Lanes 5, 8, 11, 14, 17, 20: cells with only the indicated *TLC1* allele were grown for 65 generations. Lanes 6, 9, 12, 15, 18, 21: the same cells grown for 105 generations. Terminal restriction fragments (Telo) and amplified Y′ element (arrow) are indicated on the right. (**B**) Telomere lengths in NLYH55 (*EST1*) cells expressing the indicated *TLC1* alleles and the Cdc13–Est2 fusion protein. Lane distribution is the same as above except that lanes 22–24 contain the same controls/markers as lanes 1–3.

It remained possible that *in vivo*, Est1-dependent trafficking was mediated by a different Est1–Tlc1 association than the actual recruitment of telomerase to telomeres via the Est1-Cdc13 interface. We therefore introduced the SA3, SA5 and ΔSL mutated telomerase RNAs into cells where Est1-mediated recruitment is bypassed by a Cdc13–Est2 fusion protein (32). In this strain, the forced localization of the Est2 protein, and hence the telomerase enzyme, to telomeres allows for an Est1-independent telomere maintenance (Figure 6A, lanes with WT and OPM alleles of the RNA). In contrast to the WT and OPM RNAs, none of the other mutated alleles of the Tlc1 RNA could support this form of telomerase-mediated telomere maintenance. After 105 generations of growth, all cultures assessed had switched to telomerase-independent telomere maintenance, which on a Southern blot is characterized by either amplification of a 6.7 kb Y′-element [called type I survivors (33), the ΔSL allele in lane 9, Figure 6A] or an aberrant and highly dynamic telomere banding pattern [called type II survivors (33), applies to the SA3 and SA5 alleles in Figure 6A, lanes 15, 17, 18]. In *EST1 WT* cells expressing the Cdc13–Est2 fusion and a WT Tlc1 RNA, telomeric repeat synthesis is significantly increased and telomeres become highly overelongated (32) (Figure 6B, lanes WT and OPM). Telomeres in cells harboring the Tlc1 mutant alleles in this situation, however, are significantly shorter than those in cells with the WT or OPM alleles (Figure 6B). Therefore and consistent with the *in vitro* telomerase assays (Figure 5), the ΔSL, SA3 and SA5 alleles of the Tlc1 RNA affect the level of telomerase activity on its substrate, the telomeres, in an Est1-independent fashion *in vivo*.

The *tlc1-ΔSL+IL* allele must be considered differently than the other mutant alleles because while this allele still retains the CS2 sequences, it completely lacks the CS2a sequences (37) (Figure 1). Cells harboring this allele display phenotypes as if they experienced complete loss of telomerase-mediated telomere maintenance, similar to what is seen in cells with a *tlc1Δ* allele (Figure 2B). Furthermore, no Tlc1-ΔSL+IL RNA can be detected in co-immunoprecipitation assays with Est1 (Supplementary Figure S4C) and these defects cannot be suppressed by an overexpression of Est1 (data not shown). These results suggest a dramatic reduction of the ability of this RNA to bind Est1 and hence a complete loss of telomerase-mediated telomere maintenance. However, this same ΔSL+IL RNA can be imported into the nucleus at an efficiency that is comparable with WT, even if we observed a much larger cell to cell variability with this RNA than with the other mutated versions (Figure 3A and data not shown). Therefore, while the Tlc1-ΔSL+IL RNA supports telomerase import from the cytoplasm into the nucleus, it cannot support telomerase-mediated telomere maintenance. We conclude that nuclear import of telomerase can occur even in the absence of essential Est1-binding elements.

Taken together, our findings show that the flexible architecture of the distal region on stem IVc of the telomerase RNA, a new element we call telomerase stimulating structure or TeSS, has a stimulatory role for catalytic telomerase activity. Furthermore, telomerase recruitment to telomeres requires an extended Est1-Tlc1 association that is not required for telomerase import into the nucleus.

## DISCUSSION

In an effort to elucidate molecular mechanisms of telomerase function *in vivo*, here we investigated the stem-loop IVc region in the yeast telomerase RNA Tlc1 (17,18). Previous RNA accessibility mappings using DMS (56) were by en large consistent with the predicted bulged stem-loop structure between nt 590 and 660 (18). Furthermore, co-immunoprecipitation assays as well as domain permutation experiments showed that the IVc stem-loop can fold and function as a separate domain within the Tlc1 RNA (17,27). Finally, a short conserved sequence element within this subdomain, a bulged stem around nt 647–660 called CS2, was shown to associate with the essential Est1 protein (26). However, mutations in element CS2 can be suppressed by overexpressing Est1 and an expanded phylogenetic sequence analysis uncovered an additional conserved sequence element, called CS2a, right next to CS2 (37). The potential functions of the bulged distal region of stem-loop IVc and how this subdomain affects telomerase function *in vivo* remained unknown.

Consistent with a recent report (38), we show that the CS2a element in the large bulge or IL indeed makes important contributions to Est1 binding to the RNA. This conclusion is based on the fact that deleting the complete bulged stem-loop distal to CS2 abolishes RNA co-immunoprecipitation with tagged Est1 (Supplementary Figure S4C). Moreover, cells with this Tlc1-ΔSL+IL RNA behave similarly as cells that lack telomerase altogether (Figure 2A). However, the Tlc1-ΔSL+IL RNA still supports import of telomerase from the cytoplasm to the nucleus, a process that is also dependent on Est1 [(25); Figure 3A]. These results suggest that the CS2 element on stem-loop IVc is sufficient for the nuclear import function of Est1 and that the CS2a element is not necessary for this part of telomerase trafficking. It remains to be determined whether and how the CS2–Est1 interaction is necessary for nuclear import and presently, we cannot exclude the possibility that the Est1 requirement for this step of telomerase trafficking is entirely independent of stem-loop IVc. Once inside the nucleus, however, Est1 binding to both CS2 and CS2a is required for the telomere tethering function of the Est1–Tlc1 RNA interaction. Therefore, our results suggest that Est1 contributes to telomere maintenance in multiple independent ways, consistent with the finding of multiple *est1* mutant alleles that affect telomere homeostasis in various ways (57). For example, the *est1-42* allele, which conferred a short but stable telomere phenotype and which was proposed to be deficient in a function required after telomerase recruitment (57), may be affected in the nuclear import function instead. Irrespective of what these additional various functions of Est1 may turn out to be, our results here agree with the proposition that the association of this protein with the telomerase RNA makes multiple contributions to telomerase trafficking, recruitment and activity and that these functions are separable.

Much more significantly, our results reveal that an architectural RNA element on stem-loop IVc directly affects the catalytic activity of telomerase. This element we call TeSS functions independently of the CS2 and CS2a elements. Indeed, its effect on telomerase is independent of the Est1 protein altogether. Three sets of results support this conclusion: first, specific mutations beyond the Est1 binding sites negatively affect telomere maintenance and these deficiencies cannot be overcome by overexpressing Est1 (Figures 2B and 3B). Second, in cells expressing a Cdc13–Est2 fusion protein, Est1 is not required for telomerase-mediated telomere maintenance if WT Tlc1 RNA is present. However, in the presence of the mutated Tlc1 ΔSL, SA5 and SA3, telomeres cannot be maintained by telomerase, and telomerase-independent mechanisms are used (Figure 6A). Third, relative telomerase activity on an oligonucleotide substrate assessed in cell extracts is reduced by ∼2-fold. This reduction is virtually the same in extracts derived from *EST1* or *est1Δ* cells (Figure 5). The specific alleles causing these effects either entirely lack the apical stem-loop (the *tlc1-ΔSL* allele) or cause a closing of the IL (the SA5 and SA3 alleles; Figure 1 and Supplementary Figure S2). The fact that the Tlc1-ΔSL RNA still contains the complete CS2a suggests that telomerase inhibition is not caused by a protein that binds specifically to this single stranded bulge. In support of that notion, the OPC and OPM versions of the Tlc1 RNA behave as WT in all assays, yet they contain four altered nucleotides in the internal bulge area (Figure 1 and Supplementary Figure S1). We also constructed two more *TLC1* alleles, each changing two nucleotides in the apical loop (Supplementary Figure S1), but neither of those mutations had any effect on telomerase (data not shown). It is therefore most likely that TeSS provides for a crucial interaction interface that is required for telomerase function *in vivo*. Phylogenetic sequence analyses had shown that the actual sequence of this area in *TLC1* is not conserved, even amongst the *Saccharomyces sensu stricto* group of species, yet there are three co-varying base pairs in the apical stem (starred in Figure 1A), which strongly supports structural conservation (18). Therefore, the apical stem most likely is folded *in vivo* as predicted *in silico* and it needs to be bordered by flexible unpaired nucleotides in the IL (612/613 and 633/634). We therefore propose that this RNA element, the TeSS, needs to be able to reorient in a particular fashion in the mature and active telomerase complex. This proposed induced fit model could allow TeSS to interact with and perhaps stabilize a core protein such as Est2 to remain associated with the pseudo-knot structure or the templating area of the RNA. Given that Est3 can interact with Est2 directly and some evidence suggests that it is able to assemble into an active telomerase complex without Est1, it is also possible that the core complex targeted by TeSS includes both Est2 and Est3 (58–60). Alternatively, the TeSS could need its flexibility to form an elaborate RNA structure with the pseudo-knot or neighboring sequences in the core of the enzyme. In the absence of direct evidence for these hypotheses, we cannot exclude alternate possibilities such as nonspecific inhibition of the enzyme due to an absence or mutated TeSS.

We note that the telomerase catalytic activity supported by the mutated RNAs appears not to be altered in the characteristics of nucleotide incorporation or processivity. Rather, the overall reduced patterns generated by the reactions (Figure 5) suggest that a reduced primer binding or a decreased stability of the mature elongation complex affects reaction initiation.

A long-range association in telomerase RNAs of a distal structured element with catalytic core elements is well established for other species (61,62). For example, to assemble a mature and active telomerase holoenzyme in the ciliate *Tetrahymena*, in addition to the pseudo-knot area on the RNA, a distal and flexible short stem-loop IV is required (63–65). In a conceptually related fashion, the assembly of an active mammalian TR-TERT core requires association of TERT to both the pseudo-knot area as well as the distal CR4/5 substructure on the RNA (66,67). For budding yeast, association of Est2 (yeast TERT) with the pseudo-knot area is known to be essential for enzyme function (19,27) and the results reported here strongly suggest the requirement of an additional association of the TeSS with the enzyme core. These data thus provide the first evidence for a new conserved commonality of mature telomerase complexes, namely the requirement of the catalytic core to associate with at least one additional area of the respective RNAs that is distant to the actual pseudo-knot or templating sequences.

## SUPPLEMENTARY DATA

Supplementary Data are available at NAR Online: Supplementary Tables 1–3, Supplementary Figures 1–4 and Supplementary References [68–70].

## FUNDING

Canadian Institutes for Health Research [CIHR, MOP97874 to R.J.W.]; Canadian Research Chair in Telomere Biology (to R.J.W.). Funding for open access charge: CIHR.

*Conflict of interest statement.* None declared.

## Supplementary Material

Supplementary Data
