# Supplementary material for: A new telomerase RNA element that is critical for telomere elongation
Source: Nucleic Acids Res. 2013 Jun 19;41(16):7713–24. doi: 10.1093/nar/gkt514 (PMC3763530; doi:10.1093/nar/gkt514)
Supplement: Supplementary Data [file supp_41_16_7713__index.html]

A new telomerase RNA element that is critical for telomere elongation — A new telomerase RNA element that is critical for telomere elongation — Supplementary Data 

# A new telomerase RNA element that is critical for telomere elongation

## Supplementary Data

files

**Files in this Data Supplement:**

- Supplementary Data - pdf file
