# Supplementary material for: A new telomerase RNA element that is critical for telomere elongation
Source: Nucleic Acids Res. 2013 Jun 19;41(16):7713–24. doi: 10.1093/nar/gkt514 (PMC3763530; doi:10.1093/nar/gkt514)
Supplement: Supplementary Data [file supp_gkt514_nar-01033-r-2013-File008.pdf]

SUPPLEMENTAL MATERIAL

A new telomerase RNA element that is critical for telomere elongation.

Nancy Laterreur[1], Sébastien Eschbach[2], Daniel Lafontaine[2] and Raymund J. Wellinger[1].

1. Department of Microbiology and infectious diseases

2. Department of Biology

RNA Group

Université de Sherbrooke

3201, rue Jean-Mignault

Sherbrooke, Québec, Canada

J1E 4K8

Tel: 819 821 8000 ext 75214

Fax: 819 820 6831

Address correspondence to Raymund J. Wellinger (raymund.wellinger@usherbrooke.ca)

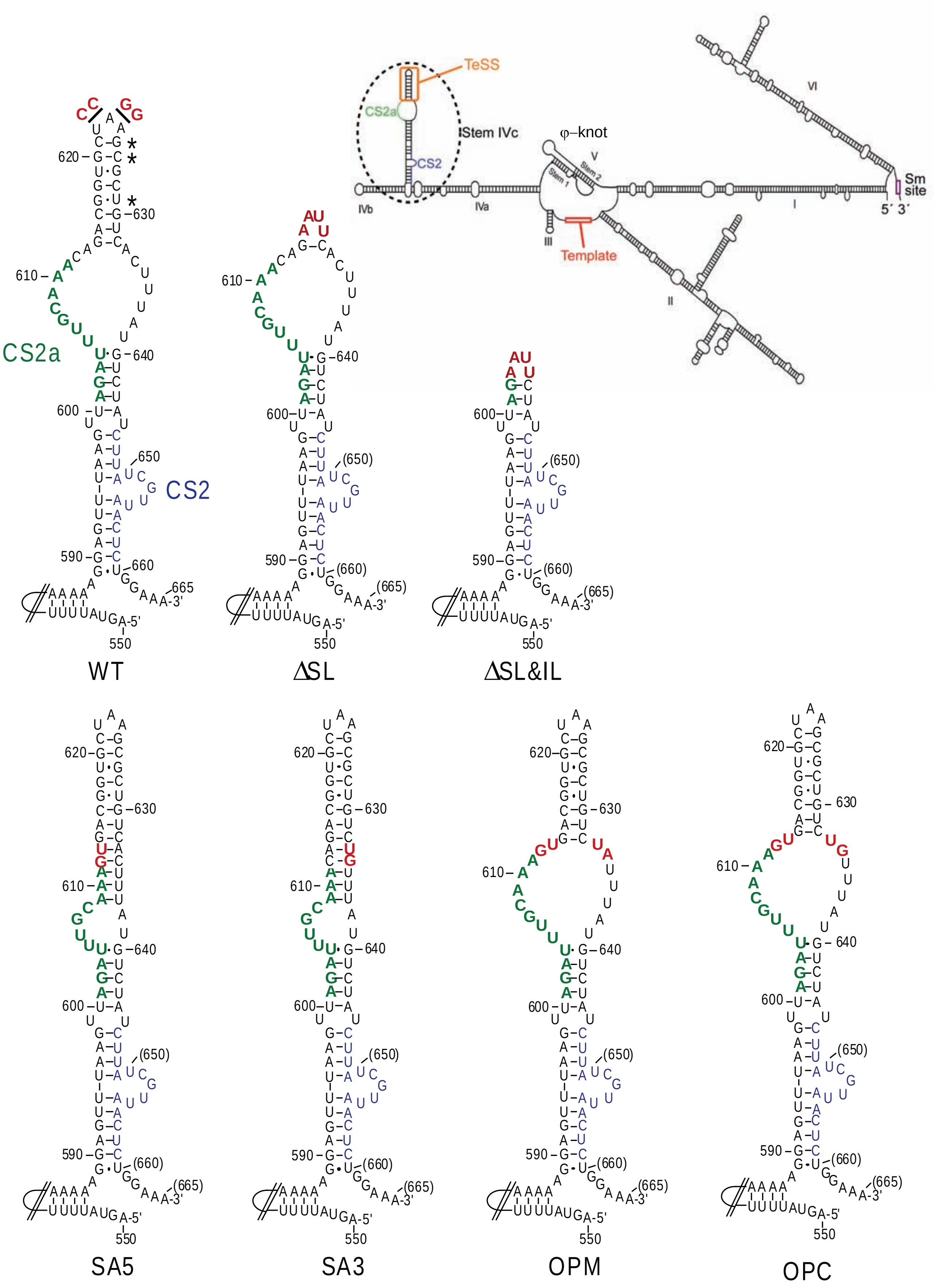
TeSS
CS2a
Stem IVc
CS2
φ−knot
V
Stem 1
Stem 2
VI
Sm site
5´ 3´
IVb
IVa
I
II
III
Template
WT
ΔSL
ΔSL&IL
SA5
SA3
OPM
OPC

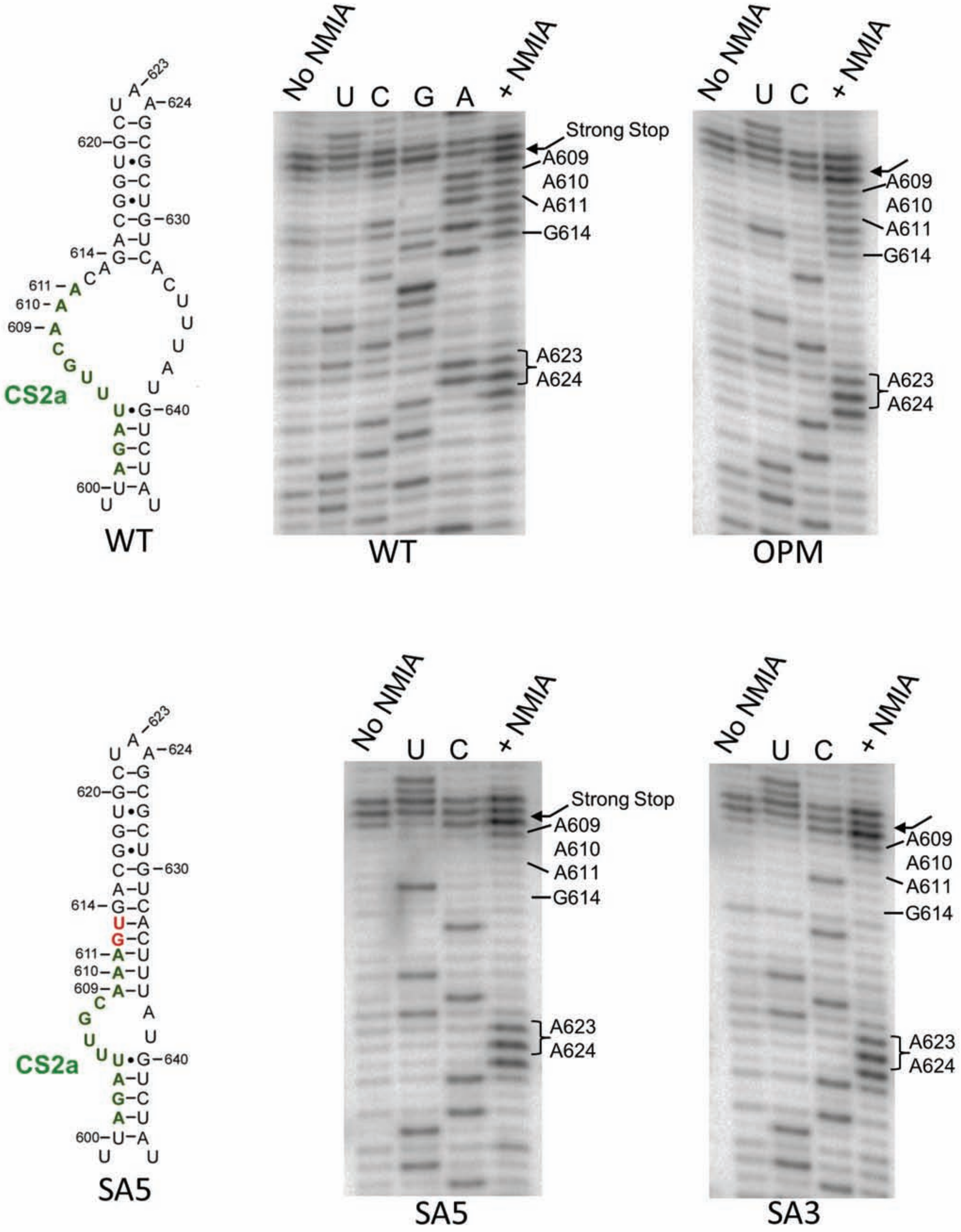

No NMIA
U
C
G
A
+ NMIA
Strong Stop
A609
A610
A611
G614
A623
A624
WT
No NMIA
U
C
+ NMIA
A609
A610
A611
G614
A623
A624
OPM
CS2a
611
610
609
614
620
623
624
630
640
600
WT
No NMIA
U
C
+ NMIA
Strong Stop
A609
A610
A611
G614
A623
A624
SA5
No NMIA
U
C
+ NMIA
A609
A610
A611
G614
A623
A624
SA3
CS2a
SA5

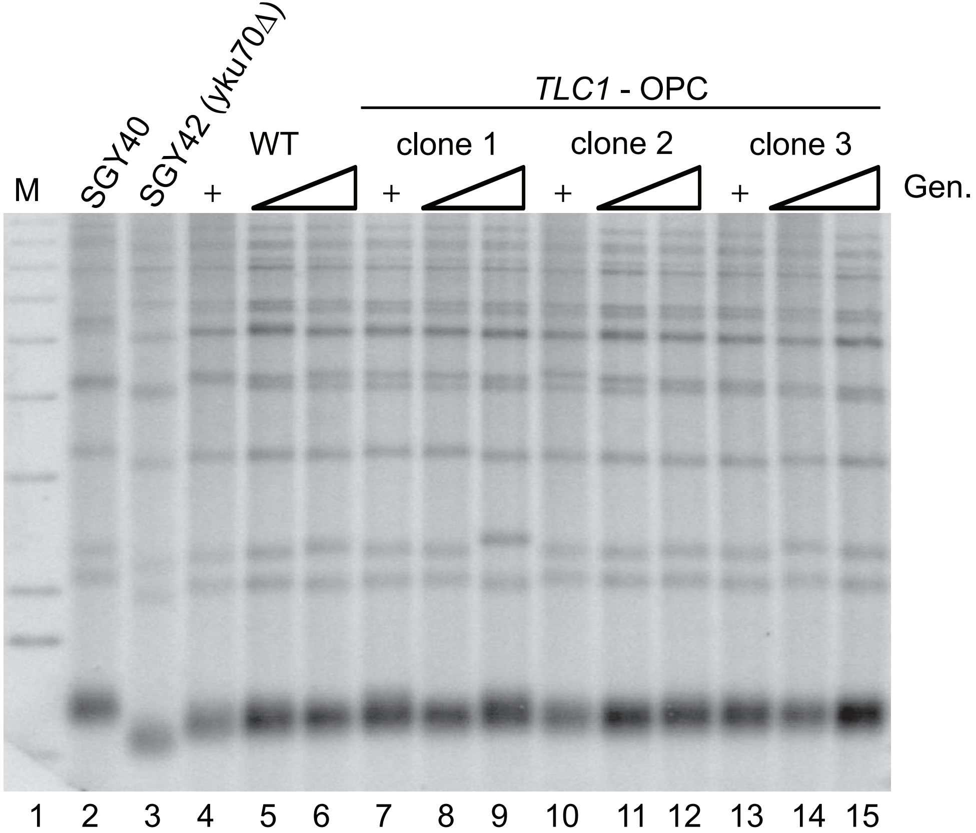
TLC1 - OPC
M
SGY40
SGY42 (yku70Δ)
WT
clone 1
clone 2
clone 3
+
+
+
+
Gen.
1
2
3
4
5
6
7
8
9
10
11
12
13
14
15

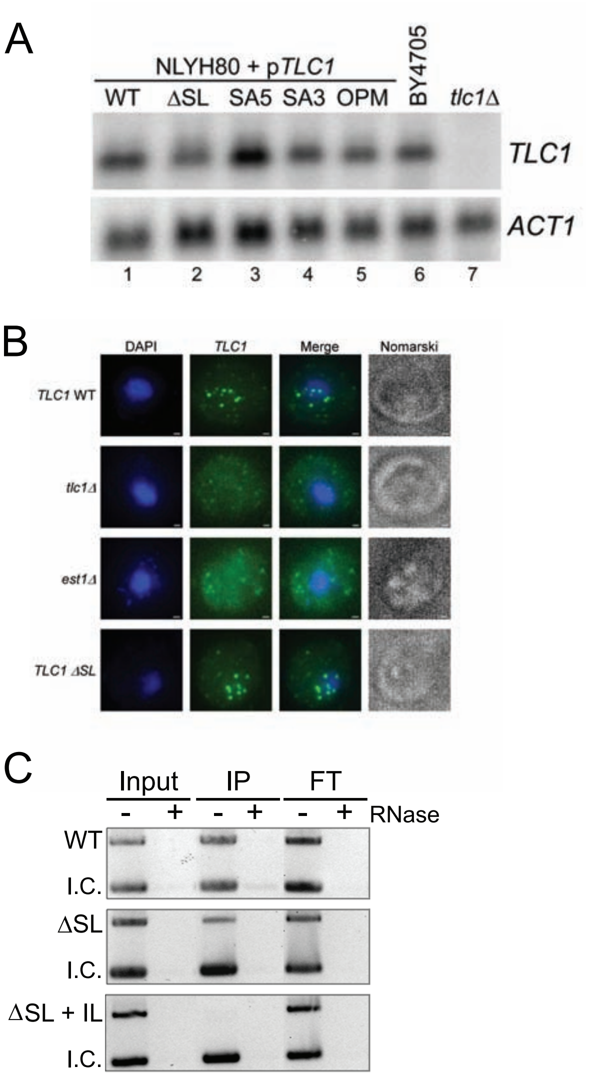
A
NLYH80 + pTLC1
WT
ΔSL
SA5
SA3
OPM
BY4705
tlc1Δ
TLC1
ACT1
1
2
3
4
5
6
7
B
DAPI
TLC1
Merge
Nomarski
TLC1 WT
tlc1Δ
est1Δ
TLC1 ΔSL
C
Input
IP
FT
- + - + - + RNase
WT
I.C.
ΔSL
I.C.
ΔSL + IL
I.C.

SUPPLEMENTARY Tables

Table S1: Yeast strains

Name	Genotype
NLYH80	*MATα ura3-52 lys2-801 ade2-101 trp1Δ63 his3Δ200 leu2Δ1 VR-ADE2-T tlc1Δ::LEU2 HA3-EST1 SME1-13MYC-KMX*
NLYH97	*MATα ura3-52 lys2-801 ade2-101 trp1Δ63 his3Δ200 leu2Δ1 VR-ADE2-T tlc1Δ::LEU2 yku70Δ::KanMX HA3-EST1 SME1-13MYC-KMX*
NLYH95	*MATα ura3-52 lys2-801 ade2-101 trp1Δ63 his3Δ200 leu2Δ1 VR-ADE2-T tlc1Δ::NatMX est1Δ::LoxP SME1-13MYC-KMX*
NLYH55	*MATα ura3-52 lys2-801 ade2-101 trp1Δ63 his3Δ200 leu2Δ1 VR-ADE2-T tlc1Δ::NatMX cdc13Δ::KanMX*
NLYH59	*MATα ura3-52 lys2-801 ade2-101 trp1Δ63 his3Δ200 leu2Δ1 VR-ADE2-T tlc1Δ::NatMX cdc13Δ::KanMX est1Δ::LoxP*
SGY40	*MATa ura3-52 lys2-801 ade2-101 trp1Δ63 his3Δ200 leu2Δ1 VR-ADE2-T*
SGY42	*MATa ura3-52 lys2-801 ade2-101 trp1Δ63 his3Δ200 leu2Δ1 VR-ADE2-T yku70Δ::LEU2*

Table S2: Plasmids

Name	Description	Reference
pAZ1	*URA3, CEN, TLC1*	(68)
pTLC1TRP	*TRP1, CEN, TLC1*	(39)
pTLC1-ΔSL	*TRP1, CEN, TLC1Δ615-630::GAATTC*	(37)
pTLC1-ΔSL+IL	*TRP1, CEN, TLC1Δ602-642::GAATTC*	(37)
pTLC1-SA5	*TRP1, CEN, TLC1 (C612G, A613T)*	This study
pTLC1-SA3	*TRP1, CEN, TLC1 (A633T, C634G)*	This study
pTLC1-OPM	*TRP1, CEN, TLC1 (C612G, A613T, A633T, C634A)*	This study
pTLC1-OPC	*TRP1, CEN, TLC1 (C612G, A613T, A633T, C634G)*	This study
pVL1107	*LEU2, CEN, CDC13-EST2*	(32)
pVL816	Integrative plasmid containing HA_3-*EST1*	(42)
pRS423-*EST1*	*HIS3*, 2 µm, *EST1*	Kind gift from K. Friedman
pSH62	*GAL-CRE*	(41)
pUG6	LoxP-KMX-LoxP cassette	(41)
p316-GAL-CRE	*URA3, CEN, GAL1-CRE*	This study
pFA6a-13Myc-KMX	13Myc-KanMX6 cassette	(43)
pAD-A258	*CEN, URA3, tlc1Δ148-440*	This study

Table S3 : Primers

Name	5´ – 3´ sequence
**PCR-mediated site-directed mutagenesis on *TLC1***	
Apical loop 1 – FOR	GCAAACAGACGGTGCCCAGCGCTGTCACTTTA
Apical loop 1 – REV	TAAAGTGACAGCGCTGGGCACCGTCTGTTTGC
Apical loop 2 – FOR	CAAACAGACGGTGCTGGGCGCTGTCACTTTATG
Apical loop 2 – REV	CATAAAGTGACAGCGCCCAGCACCGTCTGTTTG
SA5 – FOR	AGTTAGATTTGCAAAGTGACGGTGCTAAGCGCT
SA5 – REV	AGCGCTTAGCACCGTCACTTTGCAAATCTAACT
SA3 – FOR	GACGGTGCTAAGCGCTGTCTGTTTATGTCTATCTTATCGTT
SA3 – REV	AACGATAAGATAGACATAAACAGACAGCGCTTAGCACCGTC
OPM – FOR	GACGGTGCTAAGCGCTGTCTATTTATGTCTATCTTATCGTT
OPM – REV	AACGATAAGATAGACATAAATAGACAGCGCTTAGCACCGTC
**Fluorescent *in-situ* Hybridization probes (Ref. 69)** ** indicates amino-allyl modified T positions*	
TLC1-1	T*GCGCACACACAAGCAT*CTACACTGACACCAGCAT*ACTCGAAAT TCTT*TG
TLC1-2	CT*AATAAACAATT*AGCTGTAACATT*TGTGTGTGGGGT*GTGGTGA TGGT*AGGC
TLC1-3	TT*CCAGAGTTAACGAT*AAGATAGACAT*AAAGTGACAGCGCT*TAG CACCGT*C
TLC1-4	TTACGT*TCTTGATCTT*GTGTCATTGTT*CAGTTACTGAT*CGCCCGC AAACCT*
TLC1-5	TGCAT*CGAAGGCAT*TAGGAGAAGT*AGCTGTGAAT*ACAACACCAA GAT*TCA
**Selective 2'Hydroxyl Acylation analyzed by Primer Extension (SHAPE)**	
WT	CTTCCGCTTCTCTTTAGCTCCGCGAAGATAGACATAAAGTGACAGC GCTTAGCACCGTCTGTTTGCAAATCTAACTTCGCTATAGTGAGTCGT ATTACC
SA5	CTTCCGCTTCTCTTTAGCTCCGCGAAGATAGACATAAAGTGACAGC GCTTAGCACCGTCACTTTGCAAATCTAACTTCGCTATAGTGAGTCGT ATTACC
SA3	CTTCCGCTTCTCTTTAGCTCCGCGAAGATAGACATAAACAGACAGC GCTTAGCACCGTCTGTTTGCAAATCTAACTTCGCTATAGTGAGTCGT ATTAC
OPM	CTTCCGCTTCTCTTTAGCTCCGCGAAGATAGACATAAATAGACAGCGC TTAGCACCGTCACTTTGCAAATCTAACTTCGCTATAGTGAGTCGTATTA CC
T7 promo	GGTAATACGACTCACTATAGCG
**RT-PCR**	
P0 (RT)	GATCATCACGGTGCCGGATCCTTGTGTGTGGGTGTGGTGA
P1 (PCR-FOR)	TGTGCGCAATTTGTGGTTTTTTAT
P2 (PCR-Rev)	GTGATCTGCAGATCATCACGGTGCCGGATCC
***In vitro* telomerase activity assay (Ref. 70)**	
NLTAG1-3	TAGGGTAGTAGTAGGG

SUPPLEMENTARY Figure Legends

Figure S1. Schematic display of all stem IVc RNA structures.

MFold predicted secondary structures of the stem IVc arm of the Tlc1 RNA and key elements. Top right: schematic representation of the predicted secondary structure of the entire RNA. Highlights are as in Figure 1: circled region indicates location of stem IVc. Blue: CS2 element. Green: CS2a element. Stars (*): positions of co-varying base pairs. Red: positions and mutated nucleotides of the corresponding *TLC1* alleles.

Figure S2. RNA structure analyses by SHAPE.

SHAPE modification performed on *in vitro* transcribed RNA comprising the stem-loop IVc sub-structure of wild-type (WT), or mutant OPM, SA5 and SA3 sequence. First lane of each panel: reactions were performed in absence of NMIA (No NMIA). Last lane of each panel: reactions were done in presence of NMIA (+ NMIA). U, C, G and A represent sequencing lanes on the WT panel; only lanes representing U and C were loaded on the OPM, SA5 and SA3 gels. Predicted secondary structures of the WT and the SA5 mutant are shown on the left for reference. Green nucleotides: CS2a element. Red nucleotides: altered positions. Diagnostic nucleotide positions are indicated on the right of each panel. Arrow indicates “strong stop” at positions 606/607. These nucleotides, for unknown reasons, cause a strong stop in the RT-reaction, even in the absence of the NMIA compound (see left most lanes).

Figure S3. Normal telomere length in cells harbouring the *tlc1-OPC* allele.

Telomere length analysis of genomic DNA extracted from NLYH80 cells expressing WT or the *tlc1-OPC* allele. Three independent clones were tested for the OPC mutant. Lane 1 (M): end-labeled 1 Kb DNA ladder. Lanes 2 and 3: WT (SGY40) and *yku70Δ* (SGY42) controls,

respectively. Lanes 4-6: NLYH80 + p*TLC1* WT. First lane of each triplet contains DNA from cells expressing both the *tlc1-OPC* allele and the complementing WT *TLC1* plasmid pAZ1. Other two lanes of each triplet contain DNA from cells expressing only the *tlc1-OPC* allele grown for 65 and 105 generations, respectively.

Figure S4. Tlc1 RNA expression levels, its localization and co-immunoprecipitation with Est1.

(A) Top: Northern blot analysis of total RNA extracted from the NLYH80, BY4705 (WT) and *tlc1Δ* strains. Lanes 1-5 contain total RNA extracted from NLYH80 cells expressing the indicated *TLC1* alleles. Lane 6: total RNA extracted from a WT strain. Lane 7: total RNA extracted from NLYH80 that has lost the WT complementing *TLC1* plasmid pAZ1.

(**B)** Detection of the Tlc1-RNA in WT, *tlc1Δ*, *est1Δ* and *ΔSL* expressing cells by FISH assay. DAPI panels: DNA staining. *TLC1* panels: Tlc1-RNA visualized with Cy3-labeled probes (see supplementary table 3 for sequences). Merge panels: merge of DAPI and Cy3 channels. The same intensity levels were set for all except the Normarski images. Scale bar = 500 nm.

(C) RT-PCR analysis of Est1 binding to WT, ΔSL and ΔSL+IL Tlc1 RNAs. After immunoprecipitation of HA_3-Est1, RT-PCR was performed with the primers indicated in Supplementary Table 3. NLYH80 strain was transformed with both the indicated *TLC1*-expressing plasmids (WT, ΔSL and ΔSL+IL) and a smaller form of *TLC1*, *tlc1Δ148-440*, that serves as an internal control (I.C.) for Est1-binding. RNAs extracted from the input and the post-IP flowthrough (FT) were also analyzed as indicated. RNAse treatments (+ lanes) of all samples in parallel served to control for unspecific amplifications or DNA contamination.

SUPPLEMENTARY References

68. Gravel, S. and Wellinger, R.J. (2002) Maintenance of double-stranded telomeric repeats as the critical determinant for cell viability in yeast cells lacking Ku. *Mol Cell Biol*, **22**, 2182-2193.
69. Chartrand, P., Bertrand, E., Singer, R.H. and Long, R.M. (2000) Sensitive and high-resolution detection of RNA in situ. *Methods Enzymol*, **318**, 493-506.
70. Lue, N.F. and Xia, J. (1998) Species-specific and sequence-specific recognition of the dG-rich strand of telomeres by yeast telomerase. *Nucleic Acids Res*, **26**, 1495-1502.
